# Clinical effect of the internal fixation for rib fracture with single utility port complete video-assisted thoracoscopic surgery

**DOI:** 10.1186/s13019-024-02517-0

**Published:** 2024-02-05

**Authors:** Jindong Wang, Zhiguang Sun, Yongshuai Liu, Weiyong Gong, Jianxin Wang, Junyi Deng, Yue Fu, Jishan Lan

**Affiliations:** 1 The Department of Cardiothoracic Surgery, Cangzhou Integrated Traditional Chinese and Western Medicine Hospital, Cangzhou, 061000 Hebei People’s Republic of China; 2 The Department of Anesthesiology, Cangzhou Integrated Traditional Chinese and Western Medicine Hospital, Cangzhou, 061000 Hebei People’s Republic of China

**Keywords:** Thoracic trauma, Multiple rib fractures, Single utility port, Complete VATS, Internal fixation for fib fracture

## Abstract

**Backgrounds:**

The internal fixation for rib fracture with single-operation-port (two ports) complete video-assisted thoracoscopic surgery (VATS) is a promising surgical approach for treating multiple rib fractures. The study aimed to investigate the minimally invasive surgical procedure’s clinical effect in treating multiple rib fractures.

**Methods:**

Seventy-three patients with multiple rib fractures were divided into two groups according to surgical procedure. In the study group, 42 patients were operated on with the internal fixation of rib fracture with single-operation-port complete VATS. In the control group, this study performed the open operative internal fixation for rib fracture with traditional thoracotomy on 31 patients. The surgical-related indexes were retrospectively analyzed. These included the operative time, the intraoperative blood loss, the drainage amount of the chest tube, the placement time of the chest tube, the postoperative hospital stay, the incidence of postoperative complications, the imaging efficacy of rib fixation of rib fractures, and visual analog scale of pain scoring (VAS scoring).

**Results:**

There was no difference in the operative time between the study and control groups (*P* = 0.806). The intraoperative blood loss, the chest tube drainage amount, the chest tube placement time, the postoperative hospital stay, and the incidence of postoperative complications in the study group were lower than those in the control group (*P* < 0.05). There was no significant difference in the imaging efficacy of rib fixation of rib fractures between the two groups (*P* = 0.806). VAS scores in the study group on the seventh postoperative day were significantly reduced compared with the control group (*P* = 0.026).

**Conclusion:**

The internal fixation for rib fractures with single-operation-port complete VATS is a feasible, safe, simple, and minimally invasive surgical procedure to treat multiple rib fractures, which is worthy of clinical application.

## Introduction

Multiple rib fractures are the most common form of thoracic trauma, usually caused by external forces acting directly or indirectly on the chest wall. Hemopneumothorax occurs from rib fractures that injure the lung and adjacent intercostal vessels. Multiple rib fractures can cause chest wall instability or “flail chest.” In the “flail chest,” a free-floating segment of the chest wall moves paradoxically with spontaneous respiration. Severe chest wall pain results in hypoventilation and carbon dioxide retention. The combination of pulmonary contusion and chest wall pain leads to derangement in oxygenation and ventilation [1]. The open operative reduction and internal fixation treatment of multiple rib fractures with the “flail chest” have achieved satisfactory results. When open operative reduction and internal fixation of rib fractures involve multiple ribs, the traditional surgery for rib fracture reduction and fixation requires a large incision to expose multiple ribs. Chest wall muscles, blood vessels, and nerves are damaged, resulting in a high incision infection rate, chest pain, and postoperative dysfunctions, such as a limited upper limb, shoulder, and back functions and longtime numbness on the affected side of the chest [2, 3]. Therefore, the damage to muscles and nerves caused by traditional surgical methods limits the development of such techniques.

In recent years, the video-assisted thoracoscopic technique has been used for rib reduction and internal fixation of multiple rib fractures. It is applied to explore the chest cavity, repair the injured organs, and locate rib fractures, which have become necessary technical means for treating chest trauma [4–7]. However, the surgical procedure of thoracoscopy combined with a small incision performed by the traditional open-chest rib reduction and internal fixation results in damage to the structure of the chest wall and severe postoperative pain. Single-operation-port complete VATS for internal fixation of rib fractures is a promising surgical procedure for treating multiple fractures. It is based on the reduction and internal fixation of rib fractures within the chest cavity that prevents injury to the structure of the chest wall. This study investigated the feasibility and clinical effects of the internal fixation for rib fracture with single utility port complete VATS in treating multiple rib fractures by retrospective analysis.

## Materials and methods

### Clinical materials

Seventy-three patients with multiple rib fractures admitted to the hospital from June 2019 to December 2021, who conform to the inclusion criteria, were selected for the study with the approval of the hospital’s Ethics Committee. There were 54 males and 19 females, ranging from 20 to 72 years, with a mean age of 52.67 ± 19.18 years. The patients were divided into the study group (42 cases) and the control group (31 cases) according to the surgical procedure. The study group received the internal fixation for rib fractures with single-operation-port complete VATS. In contrast, the control group received the surgical open operative reduction and internal fixation for rib fractures with traditional thoracotomy. All clinical information was from the hospital medical record and the perioperative follow-up. The surgical-related index, the imaging efficacy of rib fixation of rib fractures, and the visual analog scale of pain scoring (VAS scoring) were analyzed retrospectively. The baseline demographics and characteristics of seventy-three patients with multiple rib fractures are shown in Table 1.

**Table 1:** Baseline demographics and characteristics of 73 patients with multiple rib fractures

Group	Study group (n = 42)	Control group (n = 31)	Test value	*P* value
Sex (male/female)	30/12	24/7	χ^2^ = 0.332	0.564
Age range (year)	20–72	27–70		
Mean age (year)	54.36 ± 18.65	52.18 ± 19.84	t = 1.417	0.693
Type of injury (n)			χ^2^ = 0.715	0.942
Fall injury	4	3		
Traffic injury	22	18		
Crush injury	11	8		
Other	5	2		
Number of rib fractures	4.86 ± 1.92	5.29 ± 1.92	t = 0.344	0.953
Location of rib fracture (n)				
Unilateral chest wall	31	19		
Bilateral chest wall	11	12		
Multiple injuries (n)			χ^2^ = 0.047	0.828
Yes	36	26		
No	6	5		
ISS score	8.88 ± 1.69	8.71 ± 1.32	t = 0.469	0.640

#### Inclusion criteria

(1) Preoperative chest CT showed more than two rib fractures; (2) the fracture displacement was apparent; (3) vital signs were stable; and (4) no other fatal injury.

#### Exclusion criteria

(1) Pathological fracture; (2) complicated with severe osteoporosis and severe immune system disease; (3) complicated with heart, and significant vessels injury and bronchial rupture; (4) complicated with pulmonary infection; (5) complicated with mental disorder and physical activity disorder before operation.

### Surgical procedure

This study selected the general anesthesia method, and lung isolation was used with a double-lumen endotracheal tube or bronchial blocker placed through an existing single-lumen endotracheal tube.

**Fig. 1:**
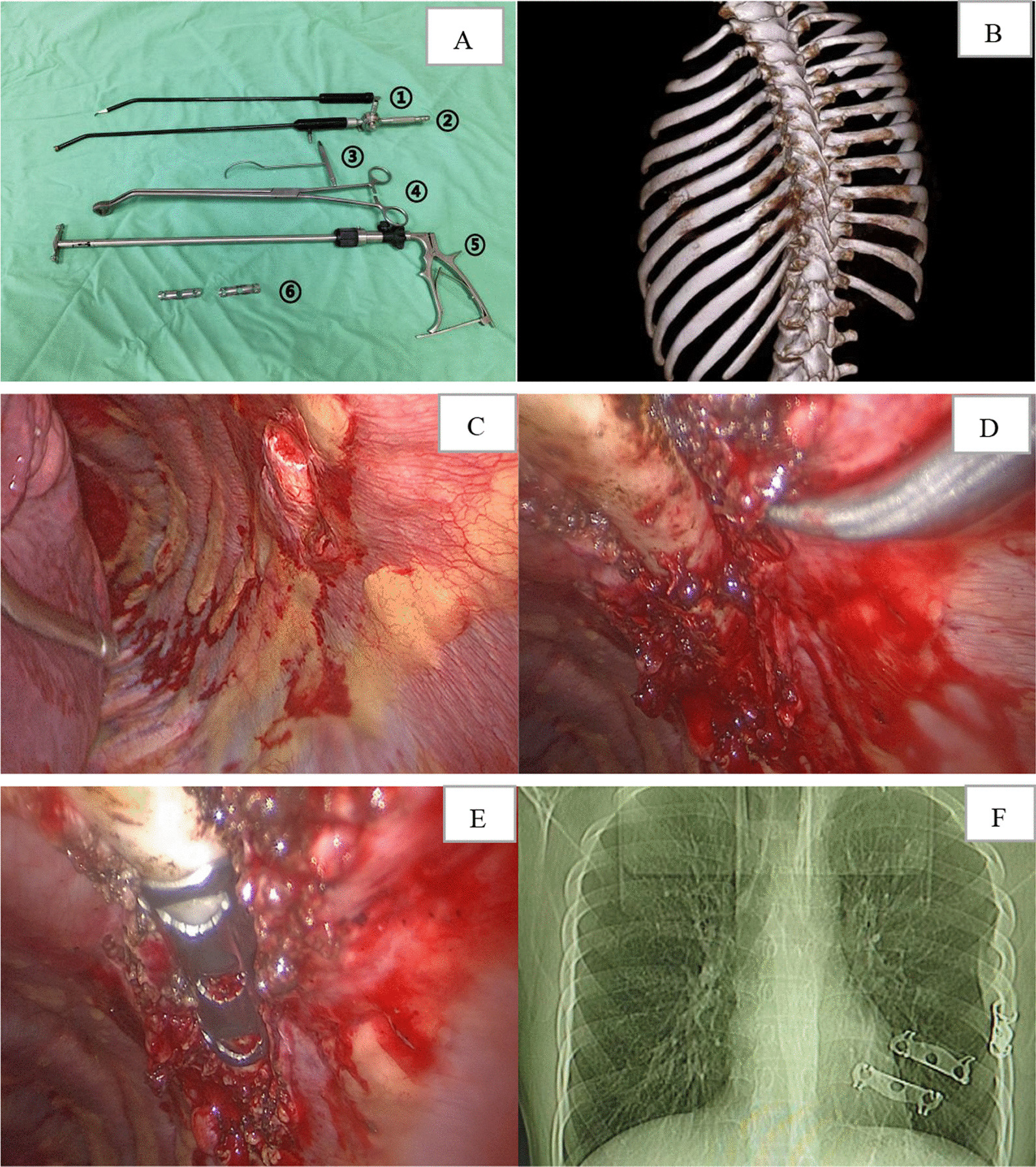
**A** The specialized instruments and the reverse memory alloy rib plate for complete VATS. ① Curving shape electro-hook, ② curving sucker, ③ puncture reduction hook, ④ “peach shape” reduction forceps, ⑤ thoracoscopic rib plate placement instrument, ⑥ reverse memory alloy rib plate. **B** A 26-year-old male patient was injured in a traffic accident. The fractures of ribs third, sixth, seventh, eighth, ninth, and 12th on the left side were revealed by chest CT and 3D reconstruction. The fractures of the sixth, eighth, and ninth ribs were obviously dislocated. Single-operation-port complete VATS for internal fixation of rib fracture was performed on the third day after chest trauma. **C** The sixth rib fracture, which was obviously displaced and punctured the pleura, was observed by thoracoscopy. **D** The sixth rib fracture was completely reduced. **E** A reverse memory alloy rib plate fixed the fractured sixth rib at the periosteum of the rib’s internal cortex. **F** Postoperative chest radiography showed that the sixth, eighth, and ninth ribs were completely reduced, and the rib plates were firmly fixed

**Fig. 2:**
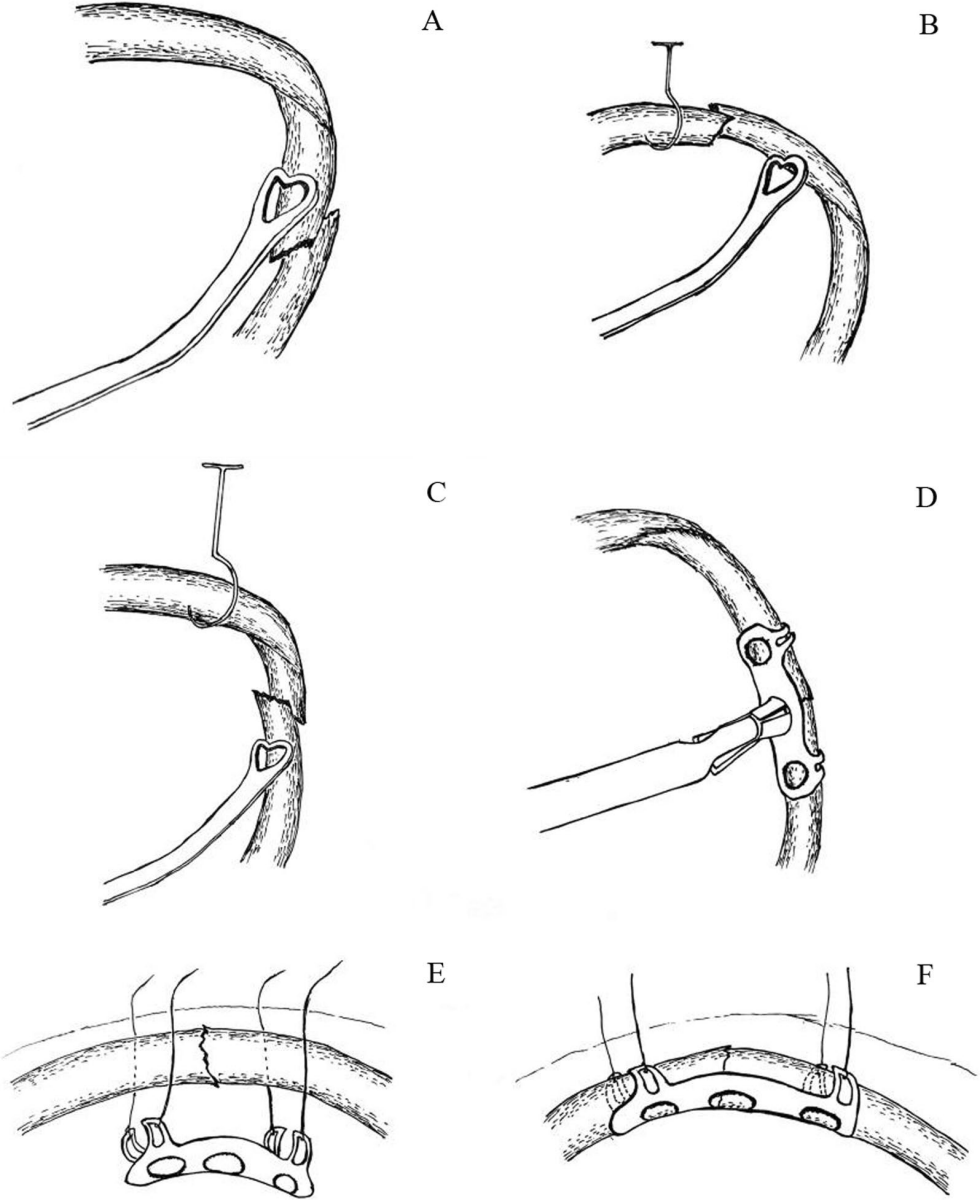
Schematic diagram of the reduction and fixation of rib fracture with single-operation-port complete VATS. **A** The distal rib of the fracture was pushed outward and upward with “peach shape” reduction forceps to achieve rib fracture reduction. **B**, **C** The puncture reduction hook penetrated the chest wall on the upper or lower rib edge at the fracture’s distal rib into the chest cavity and then rotated to hook the distal rib by pulling it upwards. The proximal end of the rib fracture was pushed outward to complete the reduction of the rib fracture combined with the “peach shape” reduction forceps. **D** The reverse memory alloy rib plate was delivered to the target rib with the help of the endoscopic rib plate placement instrument, which had a movable joint that moved forward and backward 45 degrees. It made the encircling arm’s tip of the reverse memory alloy rib plate pierce into the upper and lower edges of the fractured ribs and encircle the fractured ribs at the rib’s internal cortex. **E** A straight needle with thread was punctured 2 cm on both sides of the rib fracture line at the upper or lower edge of the rib in the thoracic cavity. The thread was pulled out through the chest wall as a traction line. The intrathoracic traction line was drawn from inside the chest along the main operating hole to the outside of the chest. The traction line bound the rib plate around the encircling arm side hole. By pulling the traction line outside the chest wall, the pre-cooled reverse memory alloy rib plate was drawn into the chest, and was placed on the target rib along the sutures pulling up. It made the tip of the encircling arm of the reverse memory alloy rib plate to pierce into the upper and lower edges of the fractured rib and **F** encircle the fractured ribs at the internal cortex of the rib

The internal fixation for rib fracture with single-operation-port complete VATS was performed on the study group. The specialized surgical instruments and reverse memory alloy rib plate for thoracoscopy are shown in Fig. 1A. The principle of small incision selection was that a certain space distance between the operative hole and the target rib facilitated the surgical instrument’s operation. A 4 cm incision in the fourth or fifth intercostal and the axillary frontline was selected as the main operative hole. A 2 cm incision in the seventh or eighth intercostal and the axillary middle line were chosen as the observation and the assisting-operative holes, respectively. The positions of incisions were adjusted according to the three-dimensional reconstruction of chest CT (Fig. 1B), combined with locating the positions of the rib fractures. This study observed the intrathoracic conditions and the positions of obvious dislocation or broken ribs of the pleura using the thoracoscopy (Fig. 1C). Obvious dislocation or broken ribs of the pleura were located. Then, hemothorax cleaning, hemostasis, and repair of lung rupture were completed under the thoracoscopy guidance. The electro-hook incised the pleura of the upper and lower edges of the rib fracture at the 4 cm end. The dislocated rib fracture was reduced using “peach shape” reduction forceps (Fig. 2A) and/or puncture reduction hook (Figs. 1D, 2B, and C). The fully cooled reverse memory alloy rib plate (TiNi encircling plate 6Z16-100, Lanzhou XiMai Memory Co., Ltd.) was delivered to the target rib with the help of a thoracoscopic rib plate placement instrument (Fig. 2D) and thoroughly drawing along sutures (Fig. 2E). This process made the tip of the reverse memory alloy rib plate’s encircling arm pierce into the upper and lower edges of the fractured ribs (Fig. 2F). The encircling arm of the reverse memory alloy rib plate was washed with sterile hot saline at 45 °C, restored to its original shape, and fixed the fractured ribs at the periosteum of the internal cortex of the rib (Fig. 1E). After flushing the chest cavity and suturing the incision, a chest tube was placed in the observation hole to drain the chest cavity.

The open-operative internal fixation for rib fracture with traditional thoracotomy was performed in the control group. According to the three-dimensional reconstruction of chest CT and ribs, combined with body surface compression, at the center of the broken and gathered area of chest ribs on the affected side, the 10–15 cm incision was made along the shape of the ribs, the skin, subcutaneous tissue. The muscles were cut layer by layer, and the fracture lines were fully exposed. Appropriate intercostals were selected, and intercostal muscles were cut. Rib retractors were used to stretch into the chest to complete hemothorax cleaning, hemostasis, and repair of lung rupture. The ribs with obvious dislocation and supporting function were selected and freed to the line of rib fracture 3 cm at both ends. The obvious dislocated rib fractures were reduced. The fully cool memory alloy plates (TiNi encircling plate, 4HLIII14-45H, Lanzhou XiMai Memory Co., Ltd.) were put on the broken position of rib fracture and washed using sterile hot saline at 45 °C. This procedure made an encircling arm of memory alloy rib plate to surround the rib at the periosteum of the external cortex of the rib and fixed for all broken ribs. At the end of the operation, a chest tube was placed to complete the operation.

### Evaluation of clinical indexes

1. Surgical-related indexes included the operative time, intraoperative blood loss, and chest tube drainage amount. It also included the placement time of the drainage tube, postoperative hospital stay, and postoperative complications (pulmonary infection, atelectasis, and pleural effusion).
2. The evaluation criteria for the imaging efficacy of rib fixation of rib fracture are as follows: Excellent: the rib is completely reduced, and the rib plate is firmly fixed. Good: ribs are slightly displaced, and the rib plate is fixed firmly. Poor: the ribs are obviously displaced, and the rib plate is not firmly fixed. The postoperative chest radiography was examined on the third or fourth day after surgery.
3. Visual analog scale of pain scoring (VAS scoring) was assessed for the degree of preoperative and postoperative chest pain before the operation and on the seventh postoperative day (the pump to relieve the pain was discontinued on the third postoperative day). VAS scoring was as follows: 0 (no pain), 10 (more severe pain), 0–2 (mild pain), 3–5 (moderate pain), 6–8 (severe pain), and 8–10 (more severe pain). The higher the score, the more severe the pain [8].

### Statistical analysis

This study used SPSS22.0 software to analyze all data. The measured data were expressed as (x ± SD) and were analyzed using the *t*-test. The enumeration data were analyzed using the chi-square test. The data of postoperative hospital stay were analyzed using the log-rank (mantel-cox) test. A *P*-value < 0.05 showed a significant statistical difference.

## Results

**Fig. 3:**
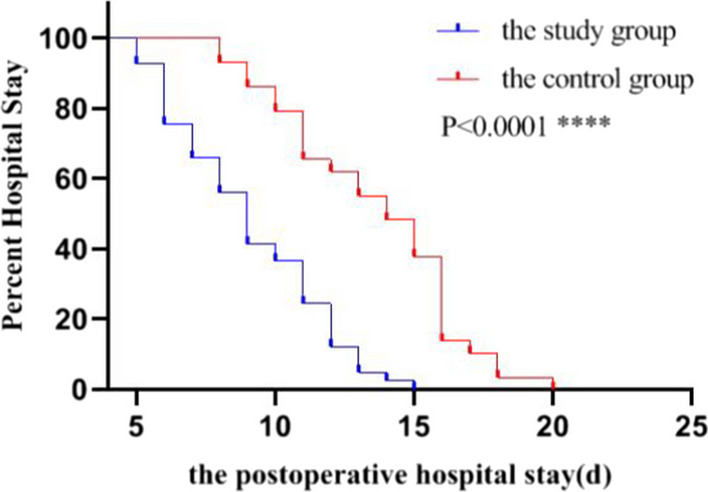
Comparison of postoperative hospital stay (d) between the study and control groups [log-rank (mantel-cox) test]

1. Baseline data of study and control groups: 73 patients with multiple rib fractures conformed to inclusion criteria. The patients were divided into study and control groups according to the surgical method. The baseline clinical variables of the two groups showed no significant difference by statistical analysis (Table 1). Therefore, the surgical-related index, the imaging efficacy of rib fixation of rib fracture and VAS score were comparable between the two groups.
2. Comparison of surgical-related indexes between the study and control groups: There was no significant difference in operative time between the two groups (*P* = 0.806) (Table 2). The intraoperative blood loss, the drainage amount of the chest tube, the placement time of the chest tube, and postoperative hospital stay in the study group were significantly lower than those in the control group (*P* < 0.05) (Table 2) (Fig. 3). There were no significant differences in the incidence of pulmonary atelectasis, pulmonary infection, and pleura effusion between the two groups (*P* > 0.05). However, the incidence of postoperative complications in the study group was significantly less than in the control group (*P* = 0.031).
3. Comparison of the imaging efficacy of rib fixation of rib fracture between the study and control groups: the chest radiography was examined on the postoperative third or fourth day (Fig. 1F). There was no significant difference in the imaging efficacy of rib fixation of rib fracture between the two groups (*P* = 0.806) (Table 3).
4. Comparison of VAS scoring between the study and control groups before operation and the seventh postoperative day: there was no significant difference in preoperative VAS scoring between the two groups (*P* = 0.371) (Table 4). However, on the seventh postoperative day, VAS scoring in the two groups was lower than before surgery (*P* < 0.05). The VAS scoring of the study group was significantly reduced compared to that of the control group on the seventh postoperative day (*P* = 0.026).

**Table 2:** Comparison of operation-relative indexes between the study and control groups

Group	Study group (n = 42)	Control group (n = 31)	Test value	*P* value
Operative time (min)	84.23 ± 21.34	87.76 ± 18.61	t = 1.033	0.806
intraoperative blood loss (ml)	65.58 ± 18.29	116.75 ± 20.06	t = 8.355	0.024
Placement time of drainage tube (d)	3.13 ± 1.09	5.87 ± 1.23	t = 7.142	0.036
Drainage amount of chest tube (ml)	102.25 ± 28.27	154.05 ± 26.81	t = 6.083	0.033
Postoperative complications			χ^2^ = 4.638	0.031
Pulmonary infection	1 (2.3%)	3 (9.6%)	χ^2^ = 1.833	0.305
Atelectasis	0 (0%)	2 (6.4%)	χ^2^ = 2.786	0.177
Pleural effusion	3 (7.1%)	4 (12.9%)	χ^2^ = 0.683	0.448

**Table 3:** Comparison of the imaging efficacy of rib fixation of rib fracture between the study and control groups

Group	n	Excellent	Good	Poor	χ^2^ value	*P* value
Study group	42	26 (61%)	14 (29%)	2 (4%)	0.302	0.860
Control group	31	21 (67%)	9 (33%)	1 (3%)

**Table 4:** VAS scoring comparison before and after surgery between the study and control groups

Group	Study group (n = 42)	Control group (n = 31)	t value	*P* value
Pre-operation	7.07 ± 2.26	7.42 ± 1.52	0.139	0.371
Postoperative 7th day	2.13 ± 1.35	4.38 ± 2.04	5.515	0.026

Comparison before with after surgery in the study group, t = 13.396, *P* = 0.008

Comparison before with after surgery in the control group, t = 6.813, *P* = 0.015

## Discussion

The treatment of rib fractures is divided into conservative and surgical treatments. In both treatments, it is crucial to maintain the stability of the chest wall. Maintaining the stability of the chest wall can reduce the degree of chest pain and restore the patient’s normal breathing [9]. Conservative treatment often uses chest band pressure bandaging, rib traction, and ventilator-assisted breathing. However, non-operative treatment has many complications, such as prolonged treatment times, obvious pain, slow healing, and other shortcomings. With the development of surgical materials and thoracic surgeons’ understanding of the disease, it has been gradually accepted that internal fixation of rib fractures as soon as possible to eliminate a series of problems caused by thoracic instability, and the advantages of surgical treatment have become increasingly prominent [10]. Some surgical indications have reached a consensus in the treatment field for multiple rib fractures [11–16].

The traditional thoracotomy with reduction and internal fixation of rib fracture has some disadvantages. It requires large incisions, causes considerable injury, has long postoperative recovery times, and offers a high incidence of postoperative complications. In recent years, video-assisted thoracoscopy has been more widely used in the emergency treatment of chest trauma. Video-assisted thoracoscopy detected the injuries of the intrathoracic lung, diaphragm, and other organs and observed the ribs with obvious dislocation. Following the site of ribs of the obvious dislocation and supporting function, a small chest wall incision was selected to repair the laceration of the lung, diaphragm, and injuries of other organs, and the complete open reduction and internal fixation of rib fracture were performed [17–20]. Although a small incision of the chest wall incision was selected, the open reduction and internal fixation of rib fracture caused damage to the chest wall muscles, nerves, and intercostal muscles, leading to chest pain and muscular dysfunction of the chest wall.

Thoracoscopy affords comprehensive visualization of the entire chest wall. The advantages of completely thoracoscopic reduction and fixation of rib fractures over extra-thoracic and combined surgical approaches may improve the exposure of rib fractures and minimize the injury to the extra-thoracic and intra-thoracic structures. Complete thoracoscopic surgery has been performed to reduce and fix rib fractures. However, it is not considered a standard surgical procedure in clinical practice due to the limitations of surgical instruments and rib-fixed materials. Su et al. [21] reported that total VATS for internal rib fixation of multiple rib fractures using implanted NiTi memory alloy rib plate and absorbable rib nail was performed on three patients. SU’s total thoracoscopic reduction and fixation of rib fracture within the thoracic cavity was technically feasible in selected patients. However, the surgical procedure was complex and unsuitable for fixing multiple rib fractures in the axillary area and scapular chest walls. Pieracci et al. [4] reported a patient with multiple rib fractures who received completely thoracoscopic intra-pleural reduction and fixation of rib fractures using a screw plate in the thoracic cavity. The advantage of surgical procedures is that the thoracic cavity can be explored while reducing and fixing the rib fractures is completed. However, the operation in the thoracic cavity is limited, and the reduction and fixation of rib fracture are difficult and has high requirements. Zhang et al. [9] reported that 31 patients with multiple rib fractures were performed with the complete VATS for internal fixation of rib fractures with a memory alloy plate within the chest cavity, achieving safe, feasible, and satisfactory results. Although the advantages of complete VATS for internal fixation of rib fractures have been detailed in treating multiple fractures, no studies have compared this surgical procedure directly to extra-thoracic surgical stabilization of rib fractures or non-operative management. Such studies will need to focus on the typical surgical-related parameters, the imaging efficacy of rib fixation of rib fracture, and postoperative chest pain.

The proposed surgical procedure was the internal fixation for rib fracture with single-operation-port complete VATS. Two small incisions were required: a 4 cm as the primary operative hole and a 2 cm as the observation and assisting-operative holes. The position of the rib fractures where the broken end of the rib fracture perforated the pleura or the subpleural hematoma occurred was identifiable under thoracoscopic observation. It was easy to expose the rib fracture end by incising the parietal pleura along the upper and lower edges of the rib. The rib was freed close to the lower edge of the rib to avoid damage to the intercostal nerves and blood vessels. For the posterior rib fractures, surgical direction from front to back, the reduction and fixation of the rib fractures were relatively easy using “peach shape” reduction forceps and the thoracoscopic rib plate placement instrument. The angle adjustment of the thoracoscopic rib plate placement instrument caused the reverse memory alloy rib plate to match the curvature and angle of the internal cortex of the rib in the chest cavity. It ensured that the reverse memory alloy rib plate was completely consistent with the shape of the target rib. For anterior and lateral rib fractures, the puncture reduction hook reduced the dislocated rib fracture, combined with the help of surgical instruments from the accessory operating hole. The pulling sutures delivered the reverse memory alloy rib plate to the target rib fracture and fixed the rib fractures at the periosteum of the internal cortex of the rib. Single utility port complete VATS for internal fixation of rib fracture thoroughly accomplished the reduction and fixation of rib fractures within the chest cavity. This minimally invasive surgical procedure avoided injuries to the chest wall and intercostal muscles, intercostal vessels, and intercostal nerves.

To the best of the authors’ knowledge, this is one of the first studies to compare the postoperative outcome of a single utility port complete VATS for internal fixation of rib fracture versus open internal fixation of rib fracture with the traditional thoracotomy. In this retrospective study, the statistical analysis showed no significant difference between the baseline clinical variables of the two groups. The analysis minimized the influence of potential selection bias between the two groups. Therefore, the two surgical techniques were comparable. Compared to the control group, the intraoperative blood loss, chest tube drainage amount, chest tube placement time, postoperative hospital stay, and postoperative complications significantly declined in the study group. These results demonstrated the advantages of the reduction and internal fixation for rib fractures with complete VATS. First, complete thoracoscopic reduction and internal fixation of rib fracture was performed to achieve rib reduction and internal fixation within the thoracic cavity. The rib was freed, and the rib end was exposed under the pleura without damaging the intercostal muscle, chest wall muscle, blood vessels, and nerves. Intraoperative bleeding was significantly decreased, and the postoperative chest pain was mild. Second, the thoracoscopic-assisted cleaning of the pleural cavity was more thorough, the pleural effusion was limited, and the thoracic drainage tube placement time was significantly shortened. VAS scoring was significantly reduced in two groups, especially in the study group. It shows that the stability of the chest wall can decline the degree of chest wall pain. The relief of chest pain and stabilization of the chest wall improved the movement of the chest wall as soon as possible, which reduced the occurrence of pulmonary infection and atelectasis.

In the study group, single-operation-port complete VATS for internal fixation of rib fracture had fewer and smaller surgical incisions, less surgical trauma, milder chest pain, and fewer complications. These advantages are associated with the patient’s quick recovery and a shorter postoperative hospital stay. The evaluation of the imaging efficacy of postoperative internal fixation for rib fractures showed no significant difference between the two groups. It suggested that the efficacy of the internal fixation for rib fracture with single-operation-port complete VATS might be comparable to open internal surgical fixation for rib fracture. Despite no difference in operative time between the two surgical procedures, developing a new surgical technique required a particular learning curve. The operative time would be significantly shortened with an increase in the number of completed surgical cases.

The internal fixation of rib fractures with single-operation-port complete VATS is a safe, feasible, and simple surgical procedure for treating multiple rib fractures. It is a less invasive surgery, causes less pain, and offers quick recovery after surgery, which complies with enhanced recovery after surgery (ERAS). The complete thoracoscopic technique is especially suitable for fixing rib fractures in the axillary, scapular, and female breast areas. These rib fractures are challenging to expose with traditional thoracotomy. This study has some limitations. Since a small sample of cases was collected in this study, statistical analysis results might be biased. More cases need to be collected for further study. The study investigated the short-term clinical effect of single-operation-port complete VATS for internal fixation of rib fracture. The long-term outcome of the minimally invasive surgical procedure in treating multiple rib fractures will be observed.

## Conclusion

The internal fixation of rib fractures with single-operation-port complete VATS is a feasible, safe, simple, and minimally invasive surgical procedure in treating multiple rib fractures, which is worthy of clinical application.

## Data Availability

All data in this study were from the patient’s medical records of Cangzhou Integrated Traditional Chinese and Western Medicine Hospital: Cangzhou, Hebei, CN. Informed consent was signed by patients before the surgery. Informed consent of patients is not required in this study, which was approved by the hospital Ethics Committee.

## References

[1] Tarng YW, Liu YY, Huang FD (2016). The surgical stabilization of multiple rib fracture using titanium elastic nail in blunt chest trauma with acute respiratory failure. Surg Endosc.

[2] Cao BX, Li Q, Lu MS (2017). Meta-analysis of open reduction internal fixation and non-operative treatment of multiple rib fractures. Chin J Trauma.

[3] Katrancioglu O, Akkas Y, Arslan S (2015). Asian Cardiovasc Thorac Ann.

[4] Pieracci FM, Johnson JL, Stovall RT (2015). Completely thoracoscopic, intra-pleural reduction and fixation of severe rib fractures. Trauma Case Rep.

[5] Zhang YC, Liu YC, Ye N (2018). Thoracoscopic assisted trans-thoracic fixation for the treatment of multiple rib fractures with hemopneumothorax. J Cardiovasc Pulm Dis.

[6] Shang Y, Gao HM, Li ZW (2016). Clinical efficacy of video assisted thoracic traditional transthoracic fixation for multiple rib fractures combined with hemopneumothorax. Med Recapitulate.

[7] Wang DD, Xu YD, Wang QQ (2022). A cohort study on the comparison of complications, short-term efficacy, and quality of life between thoracoscopic surgery and traditional Surgery in the treatment of rib fractures. Contrast Media Mol Imaging.

[8] Boonstra AM, Schiphorst Preuper HR, Balk GA (2014). Cut-off points for mild, moderate, and severe pain on the visual analogue scale for pain in patients with chronic musculoskeletal pain. Pain.

[9] Zhang JJ, Hong QC, Muo XC (2020). Use of patented thoracoscopic rib plate in total video-assisted thoracoscopic memory alloy internal fixation for rib fractures. Chin J Min Inv Surg.

[10] Li SM, Li JG, Li Q (2021). Effect of minimally invasive plate internal fixation on postoperative pain, respiratory function and fracture healing in elderly patients with rib fracture. Chin J Gerontol.

[11] Qiao GB, Chen G (2018). The management of traumatic rib fractures: Guangdong thoracic surgical consensus (2017 edition). Chin J Clin Thorac Cardiovasc Surg.

[12] Kong LW, Huang GB, Yi YF, Du DY; Consensus expert group. The Chinese consensus for surgical treatment of traumatic rib fractures 2021 (C-STTRF 2021). Chin J Traumatol. 2021; 24:311–19. 10.1016/j.cjtee.2021.07.012 PMC8606596 34503907

[13] Pieracci FM, Majercik S, Ali-Osman F (2017). Consensus statement: surgical stabilization of rib fractures rib fracture colloquium clinical practice guidelines. Injury.

[14] Bemelman M, de Kruijf MW, van Baal M (2017). Rib fractures: to fix or not to fix? An evidence-based algorithm. Korean J Thorac Cardiovasc Surg.

[15] Farquhar J, Almarhabi Y, Slobogean G (2016). No benefit to surgical fixation of flail chest injuries compared with modern comprehensive management: results of a retrospective cohort study. Can J Surg.

[16] Uchida K, Nishimura T, Takesada H (2017). Evaluation of efficacy and indications of surgical fixation for multiple rib fractures: a propensity-score matched analysis. Eur J Trauma Emerg Surg.

[17] Zhu W, Xiu ZG, Hui Y (2019). A clinical analysis of complete video-assisted thoracoscopic surgeries for management of multiple fractured ribs with thoracic traumas. Chin J Thorac Surg (Electron Ed).

[18] Ju YJ, Yin CL, Chen XY (2016). Comparative analysis on thoracoscopic surgery and conventional thoracotomy for emergency surgical treatment in multiple rib fracture complicating pulmonary laceration. ChongQing Med.

[19] Fu HB, Kong XH, Wei HB (2019). Internal fixation surgery of fractures under single-port thoracoscopy. Chin J Thorac Surg (Electron Ed).

[20] Luo H, Peng JM, Yuan YE (2020). Clinical effect of thoracoscopic internal fixation in treatment multiple rib fractures and analysis of the ameliorative effect of pulmonary ventilation disorder. Hebei Med.

[21] Su ZY, Zhang YL, Wei F, Jiang TS (2013). Clinical application of SU, total VATS rib fracture fixation technique using bone plate and bone nail. Chin J Clin Thorac Cardiovasc Surg.

